# Supplementary material for: C. elegans monitor energy status via the AMPK pathway to trigger innate immune responses against bacterial pathogens
Source: Commun Biol. 2022 Jun 30;5:643. doi: 10.1038/s42003-022-03589-1 (PMC9246835; doi:10.1038/s42003-022-03589-1)
Supplement: Supplementary file 2 — Supplemental Information [file 42003_2022_3589_MOESM2_ESM.pdf]

Supplementary Material for
"*C. elegans* monitor energy status via AMPK pathway to trigger innate immune responses against bacterial pathogens"

Shouyong Ju[#, 1], Hanqiao Chen[#, 1], Shaoying Wang[1], Jian Lin[1], Yanli Ma[1], Raffi V Aroian[2], Donghai Peng[*, 1], Ming Sun[*, 1]

1. State Key Laboratory of Agricultural Microbiology, Hubei Hongshan Laboratory, National Engineering Research Center of Microbial Pesticides, Huazhong Agricultural University, Wuhan 430070, China.

2. Program in Molecular Medicine, University of Massachusetts Chan Medical School Worcester, Worcester MA 01605-2377, USA.

# These authors contributed equally to this work.

* These authors jointly supervised this work. To whom correspondence: Tel: 86-27-87283455; Fax: 86-27-87280670; E-mail: m98sun@ mail.hzau.edu.cn & donghaipeng@mail.hzau.edu.cn

Supplementary Figures:

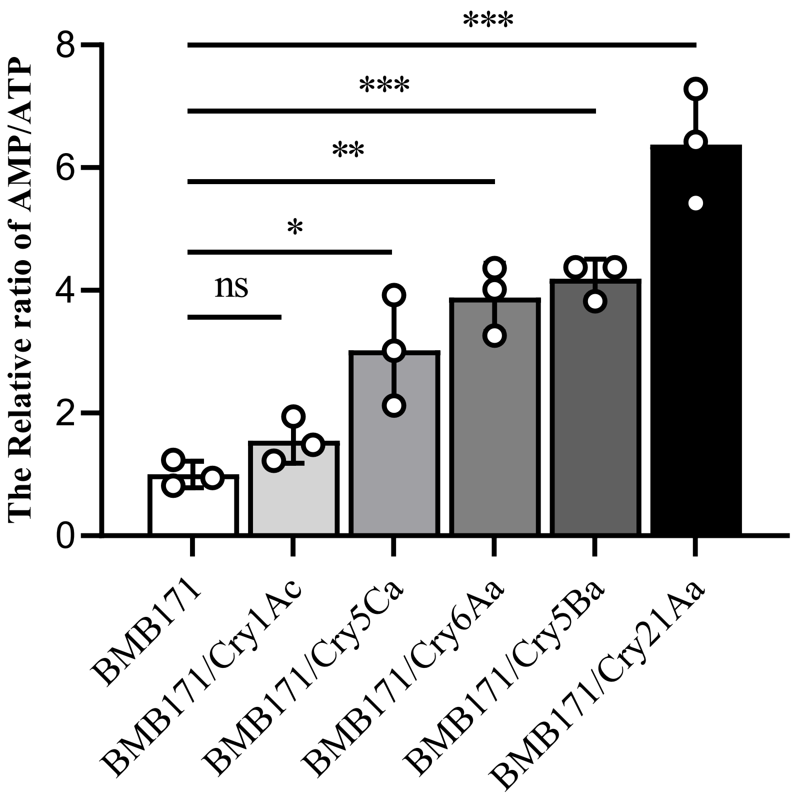

Supplementary Figure 1. Nematocidal Bt strains cause energy disturbance in *C. elegans*. *C. elegans* N2 were fed with nematocidal *B. thuringiensis* BMB171/Cry5Ca, BMB171/Cry21Aa, BMB171/Cry6Aa, and a non-nematocidal *B. thuringiensis* BMB171/Cry1Ac. The AMP/ATP ratio were then analyzed by LC-MS/MS. N =3 independent experiments. Data points represent the mean values of three independent replicates, error bars denote the SD. Values differences were determined by one way ANOVA with Dunnett's test. ***: p <0.001, **: p <0.01, *: p < 0.05.. ns indicate no significant difference.

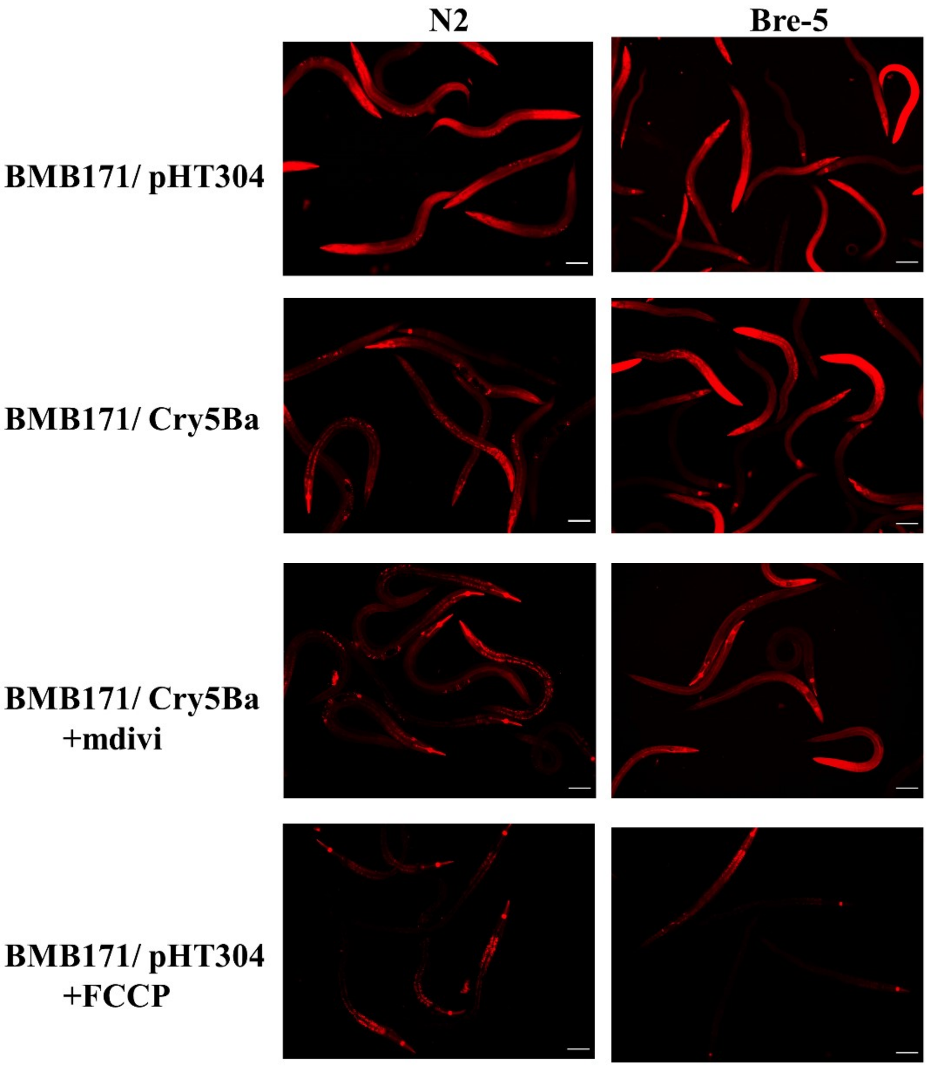

Supplementary Figure 2. BMB171 / Cry5Ba infection reduces the mitochondrial membrane potential (ΔΨm) of *C. elegans*. *C. elegans* N2 and *bre-5* were fed BMB171 / pHT304 and BMB171 / Cry5Ba respectively. Mdivi-1 were used to inhibit mitochondrial MF and FCCP was used as a positive control to reduce mitochondrial membrane potential. Treated animals were incubated in TMRE and observed microscopically. The scan bars represent 100 μm. Representative images of worms in each condition are shown. N =3 independent experiments.

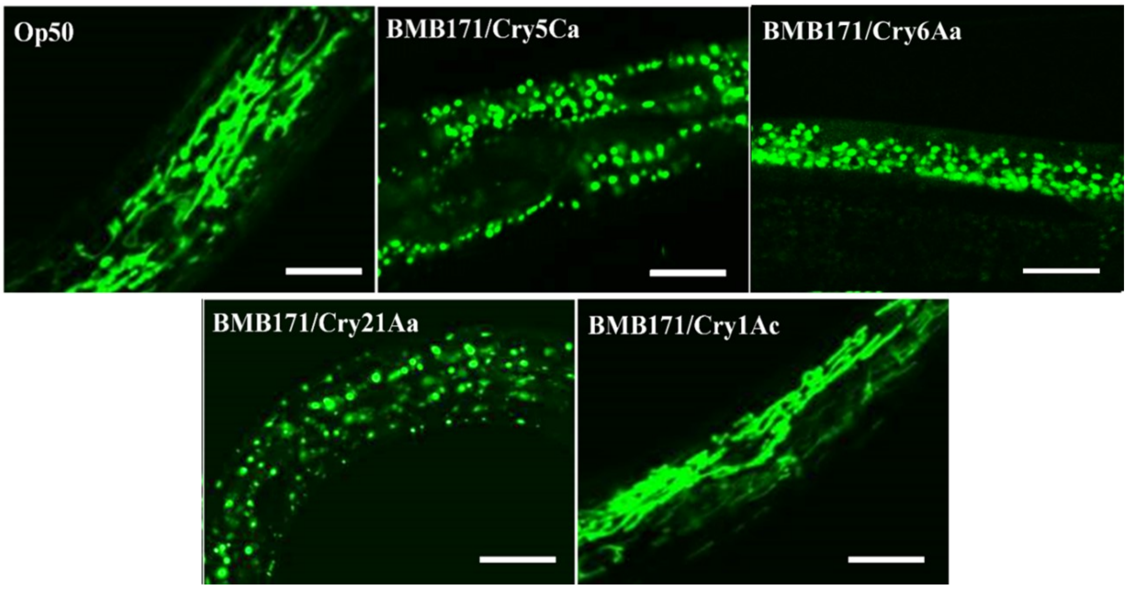

Supplementary Figure 3. Nematocidal Bt strains that produce Cry5-like Cry proteins cause MF phenomenon in *C. elegans*. The transgenic *C. elegans* SJ4143(*zcIs17* [$P_{ges\text{-}1}$::GFP^{mt}]) were fed with nematocidal *B. thuringiensis* BMB171/Cry5Ca, BMB171/Cry21Aa, BMB171/Cry6Aa, and a Non-nematocidal *B. thuringiensis* BMB171/Cry1Ac. The mitochondria morphologies of transgenic *C. elegans* were observed after 4 h of treatment. More than 60 worms were observed for each treatment and the representative images are shown. These strains are the recombinant *B. thuringiensis* which producing Cry5Ca, Cry21Aa, Cry6Aa, and Cry1Ac proteins in the acrystalliferous *B. thuringiensis* strain BMB171 respectively. The *E. coli* strain OP50 was used as negative control. The scan bars represent 20 μm. N =3 independent experiments.

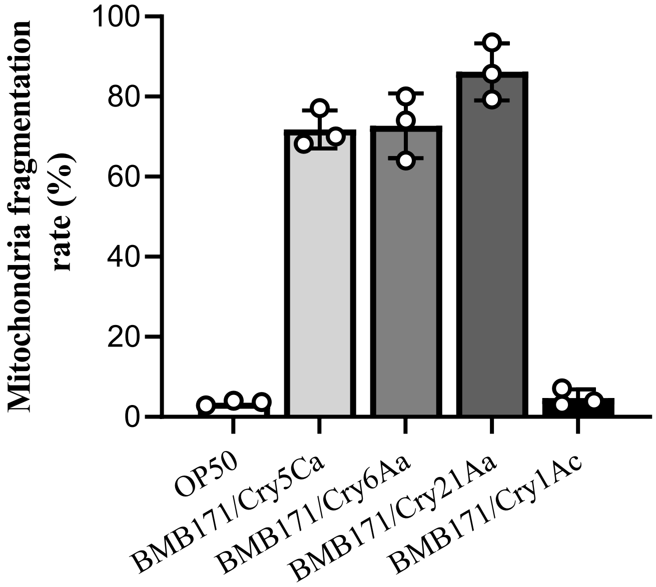

Figure S4. Analysis of the percentage of worms that showed MF phenotype after treatment with different strains. More than 60 worms were observed for each treatment. N =3 independent experiments. Data points represent the mean values of three independent replicates, error bars denote the SD. Values differences were calculated by one way ANOVA with Dunnett's test. ***p< 0.001 versus control (OP50). N =3 independent experiments

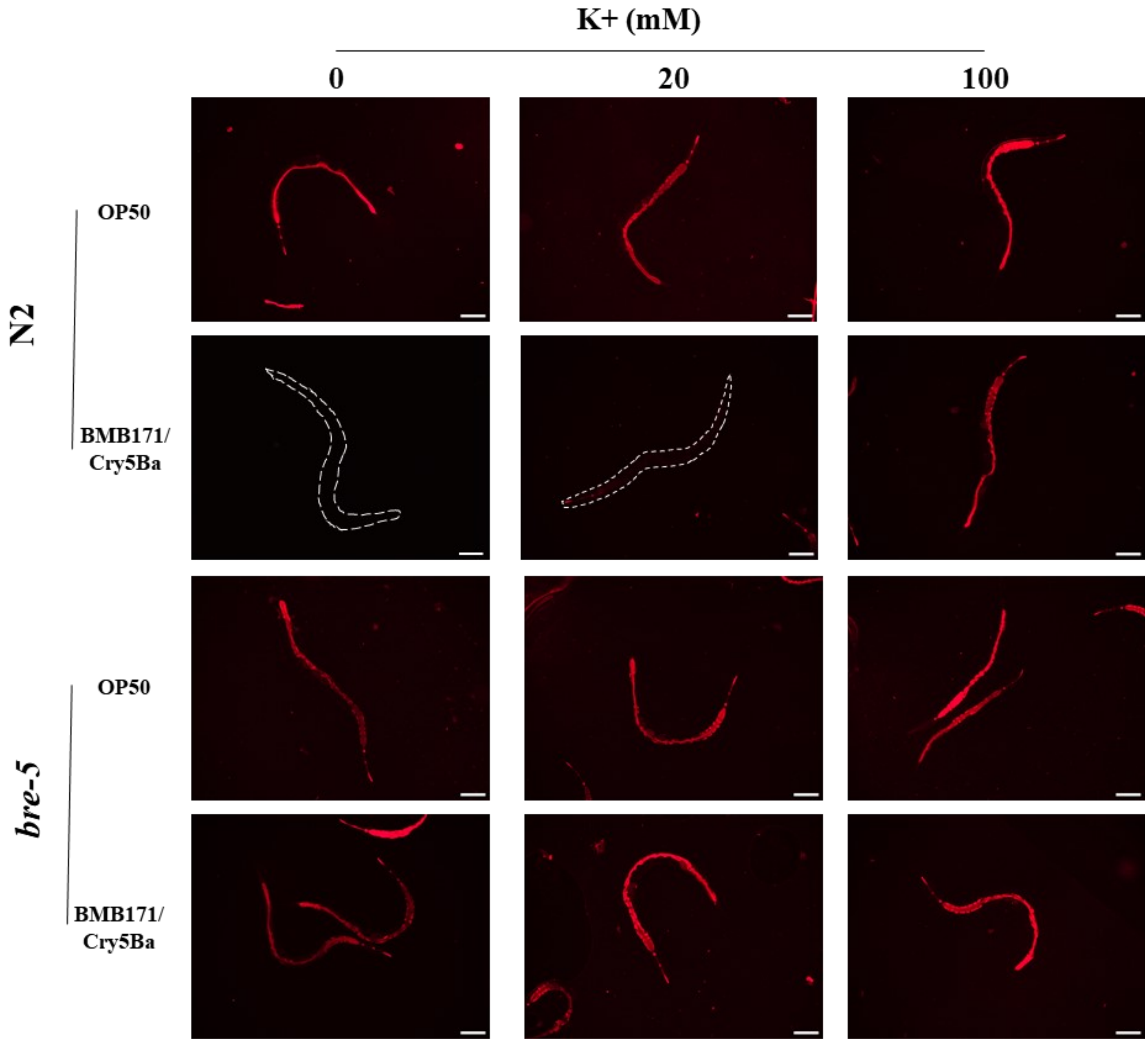

Supplementary Figure 5. Visual observation the potassium concentration during BMB171/Cry5Ba infection. *C. elegans* N2 and *bre-5* (*ye17*) were fed with OP50 or BMB171/Cry5Ba during different potassium environment. Fluorescence microscopy was used to monitor potassium concentration by measuring cytoplasmic fluorescence using the APG-2 AM. The representative images of worms treated by each strain are shown. Scale bar, 100 µm. N =3 independent experiments.

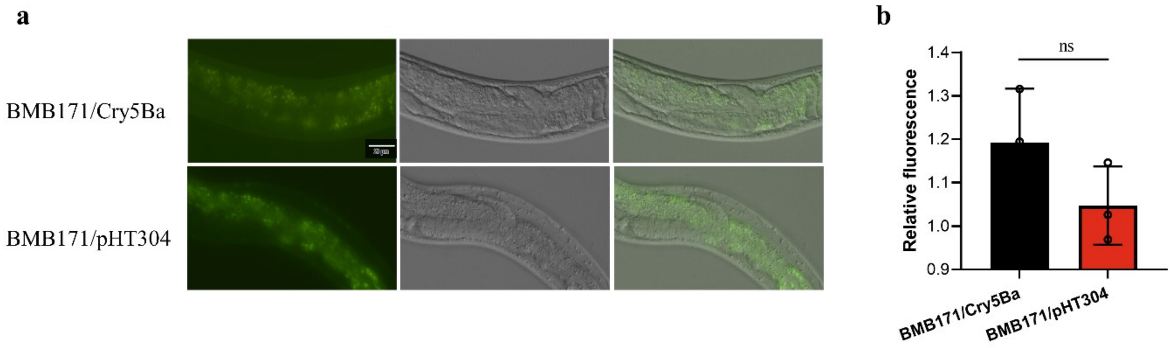

Supplementary Figure 6. Visual observation the cytoplasm calcium concentration after BMB171/Cry5Ba infection in *C. elegans*. Fluorescence microscopy was used to monitor calcium concentration by measuring cytoplasmic fluorescence using the calcium indicator Fluo-4 AM. The below part shows the quantification of the fluorescence levels. Data points represent the mean values of three independent replicates, error bars denote the SD. Scale bar, 0.02 mm. The p value was determined by a student's t-test (Welch's correction for unequal variances). Ns indicate no significant difference. Representative images of worms in each condition are shown. N =3 independent experiments.

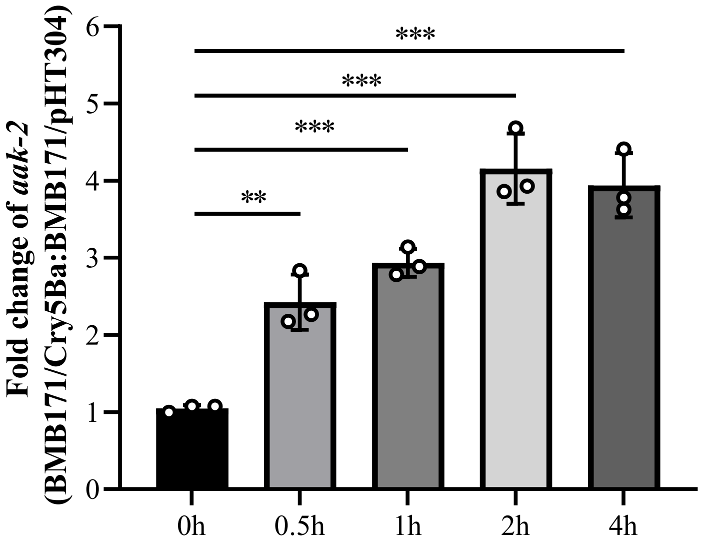

Supplementary Figure 7. The transcription levels of *aak-2* after Bt infection. QRT-PCR to detect the transcription levels of *aak-2* of wild type N2 worms when fed with BMB171/Cry5Ba and BMB171/pHT304. N =3 independent experiments. Data points represent the mean values of three independent replicates, error bars denote the SD. Values differences were calculated by one way ANOVA with Dunnett's test. ***: p <0.001, **: p <0.01.

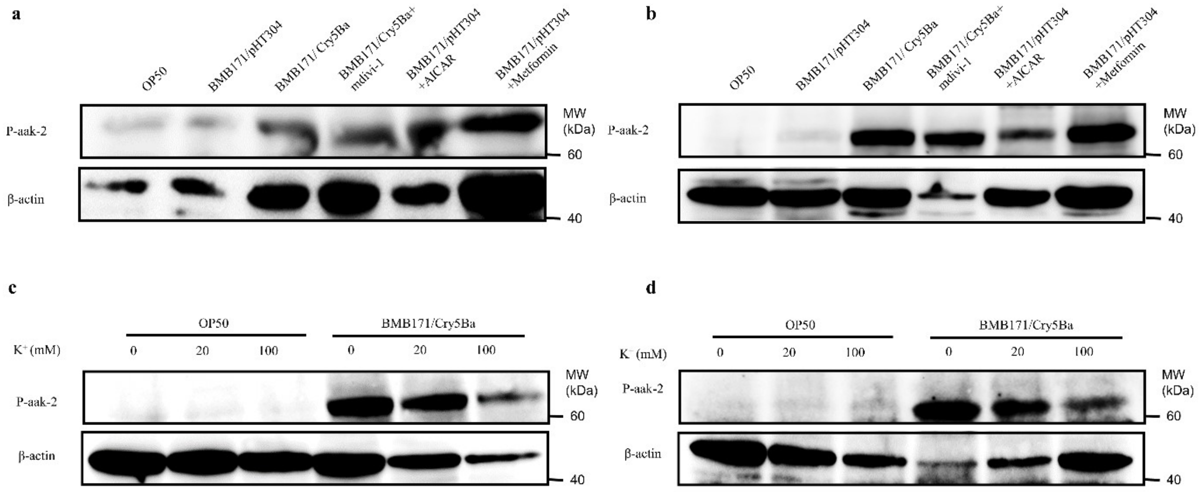

Supplementary Figure 8. Western blotting detect the activation of AMPK by the phosphorylation of the protein AAK-2. Western blotting showed the phosphorylation of the protein AAK-2 when worms exposed to OP50, BMB171/pHT304 and BMB171/Cry5Ba mixed with mdivi-1, AICAR and metformin or not in **a** and **b**. The phosphorylation of the protein AAK-2 after worms fed with OP50 and BMB171/Cry5Ba in different potassium environments showed in **c** and **d**.

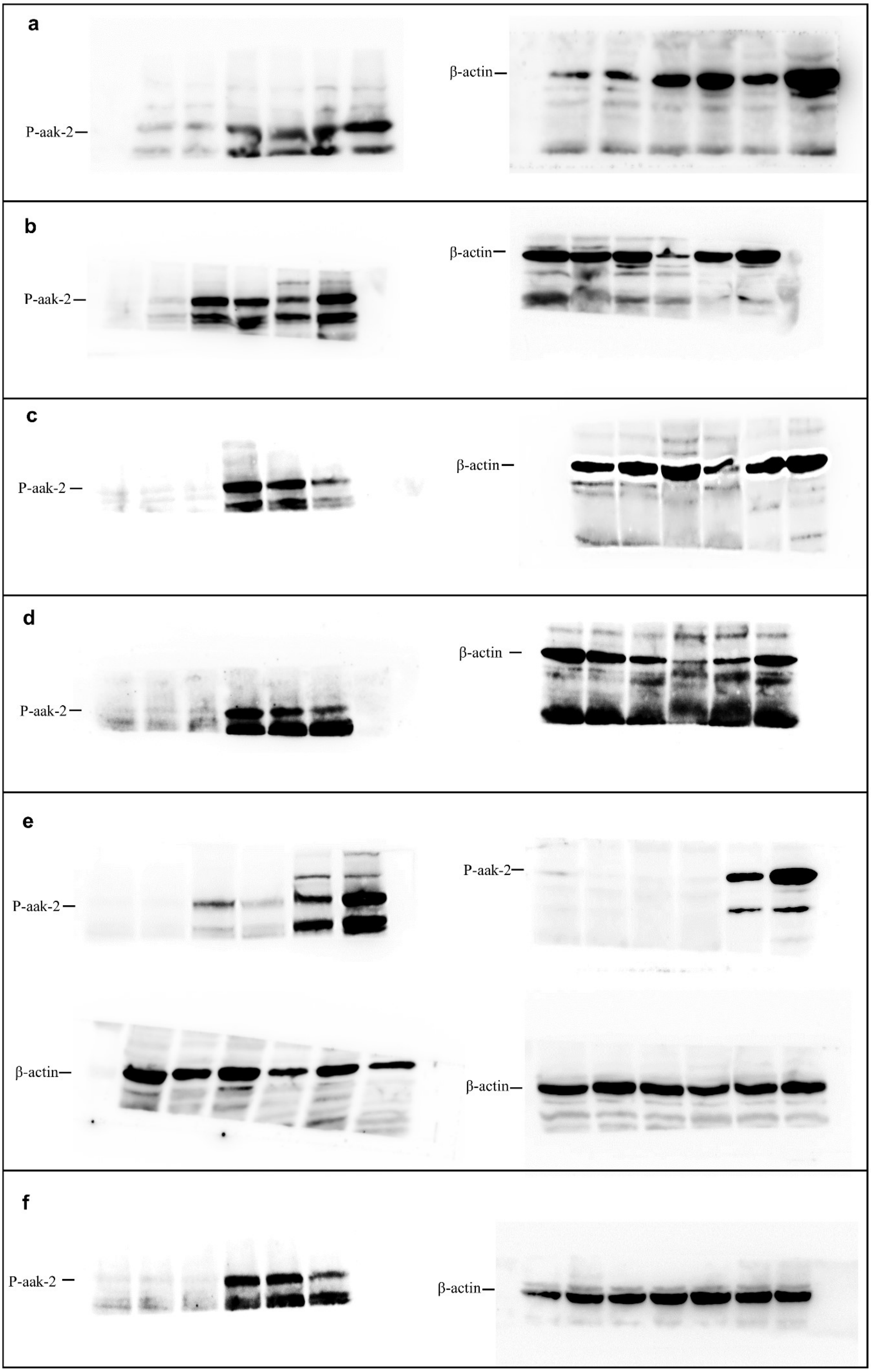
a
P-aak-2
β-actin
b
P-aak-2
β-actin
c
P-aak-2
β-actin
d
P-aak-2
β-actin
e
P-aak-2
P-aak-2
β-actin
β-actin
f
P-aak-2
β-actin

Supplementary Figure 9. Uncropped and unedited Western Blotting images. The figure a-d represent each of the Western Blotting images related to figure a-d in Supplementary Figure 8, whereas figures e-f are corresponded to Figure 4a and Figure 4c.

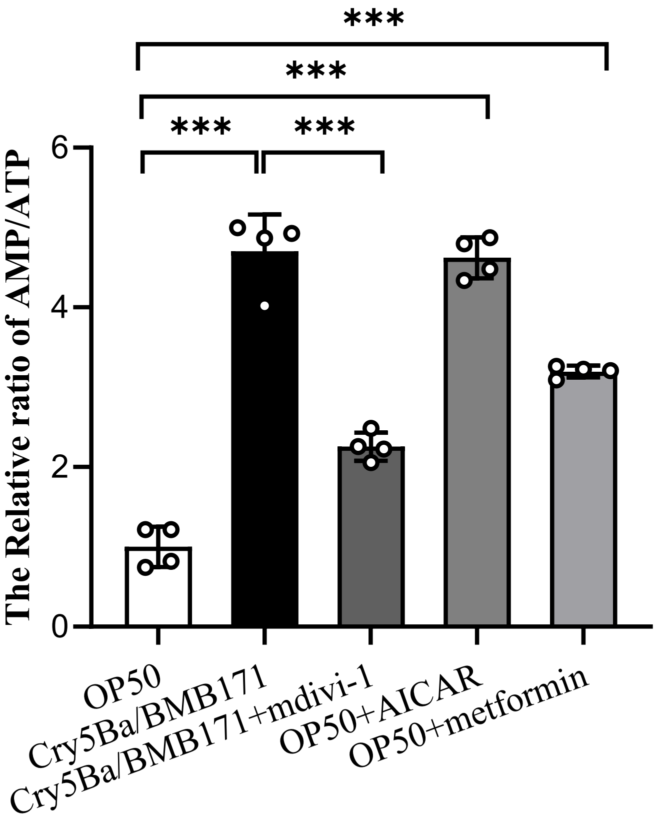

Supplementary Figure 10. The relative AMP/ATP ratio after BMB171/Cry5Ba infection or added with mdivi-1, AICAR and metformin. N =3 independent experiments. Data points represent the mean values of three independent replicates, error bars denote the SD. The p value was determined by a Student's t-test (Welch's correction for unequal variances). ***: *p* <0.001.

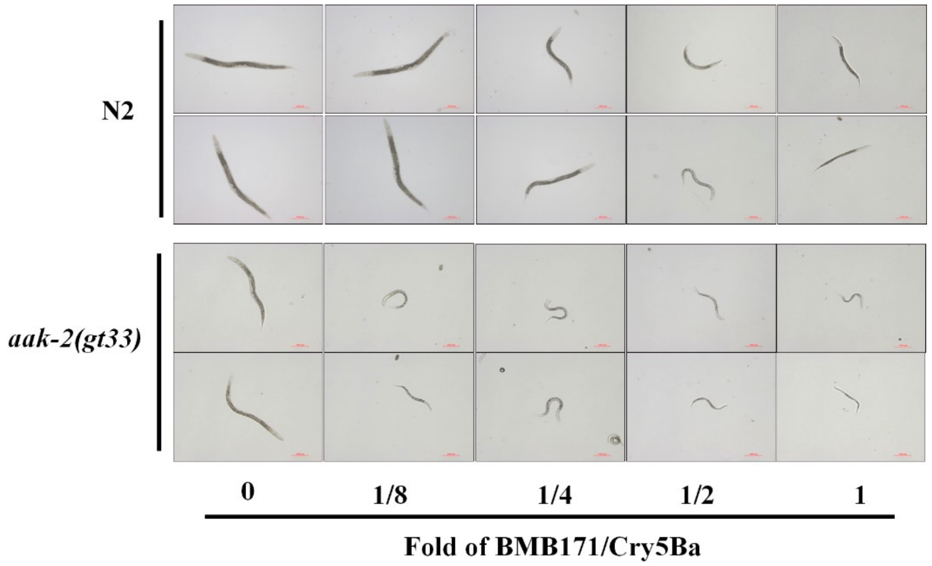

Supplementary Figure 11. The plates assay revealed *aak-2* mutant *aak-2 (gt33)* worms are more sensitive to BMB171/Cry5B infection. The growth assay of the wild type N2 and mutant worms after BMB171/Cry5Ba infection. A total of 100-200 L1-staged worms were exposed to each dose of BMB171/Cry5Ba in plates for 3 days. The experiment was performed at least three independent replicates and the representative worms are shown for each dose. Scale bar, 0.02 mm. N =3 independent experiments.

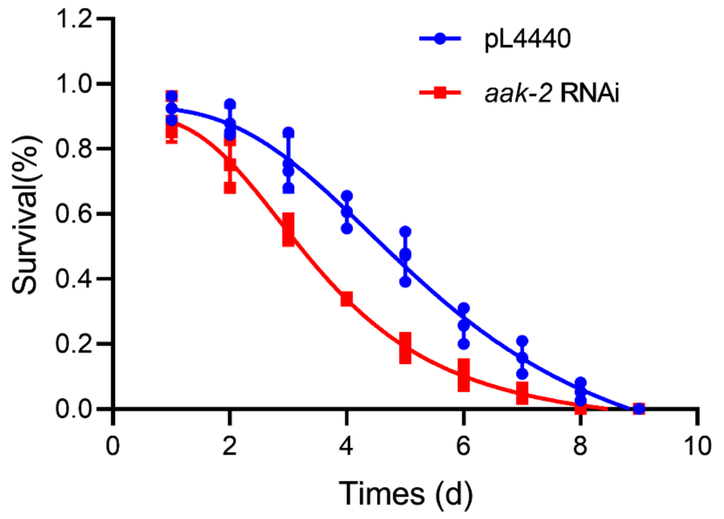

Supplementary Figure 12. A mortality assay revealed *aak-2* RNAi worms are more hypersensitive to BMB171/Cry5B infection. The survival rate of the wild type worm N2 with empty vector and *aak-2* RNAi worms exposed to BMB171/Cry5Ba at different times were recorded. A total of 30~50 L4 stage synchronized worms were scored for survival per replicate. N =3 independent experiments. Data points represent the mean values of three independent replicates, error bars denote the SD. $p<0.01$ relative to N2 control with empty vector (pL4440).

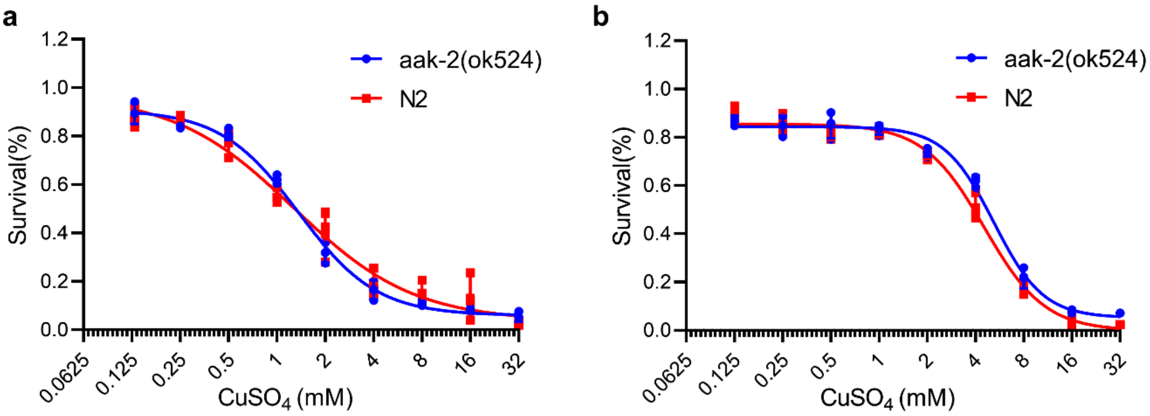

Supplementary Figure 13. A dose-dependent mortality assay revealed aak-2 mutants are not hypersensitive to chemical toxins insult compared to wild-type N2. a. The survival assay of the wild worm N2 and null allele *aak-2* mutant *aak-2 (ok524)* in different doses of heavy metal $CuSO_4$. b. The survival assay of the wild worm N2 and null allele *aak-2* mutant *aak-2 (ok524)* in different doses of oxidative stress agent H_2O_2. A total of 20~30 L4 stage synchronized worms were scored for mortality per replicate. The mortality was determined after 3 days for Bt strains or $CuSO_4$ exposure, and 4 h for H_2O_2 exposure. N =3 independent experiments. Data points represent the mean values of three independent replicates, error bars denote the SD. No significant difference was relative to N2 control.

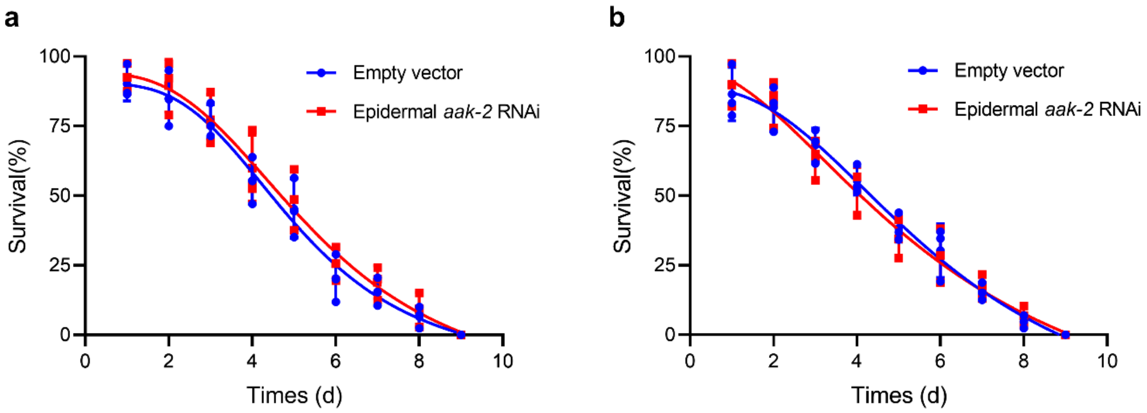

Supplementary Figure 14. Mortality assay revealed epidermal (a) or muscular-specific (b) *aak-2* RNAi had no effect on worm's sensitivity to Bt infection. The survival assay of the wild worm N2 and RNAi worms exposed to BMB171/Cry5Ba at different times were recorded. A total of 20~30 L4 stage synchronized worms were scored for mortality per replicate. N =3 independent experiments. Data points represent the mean values of three independent replicates, error bars denote the SD. No significant difference was relative to empty vector controls.

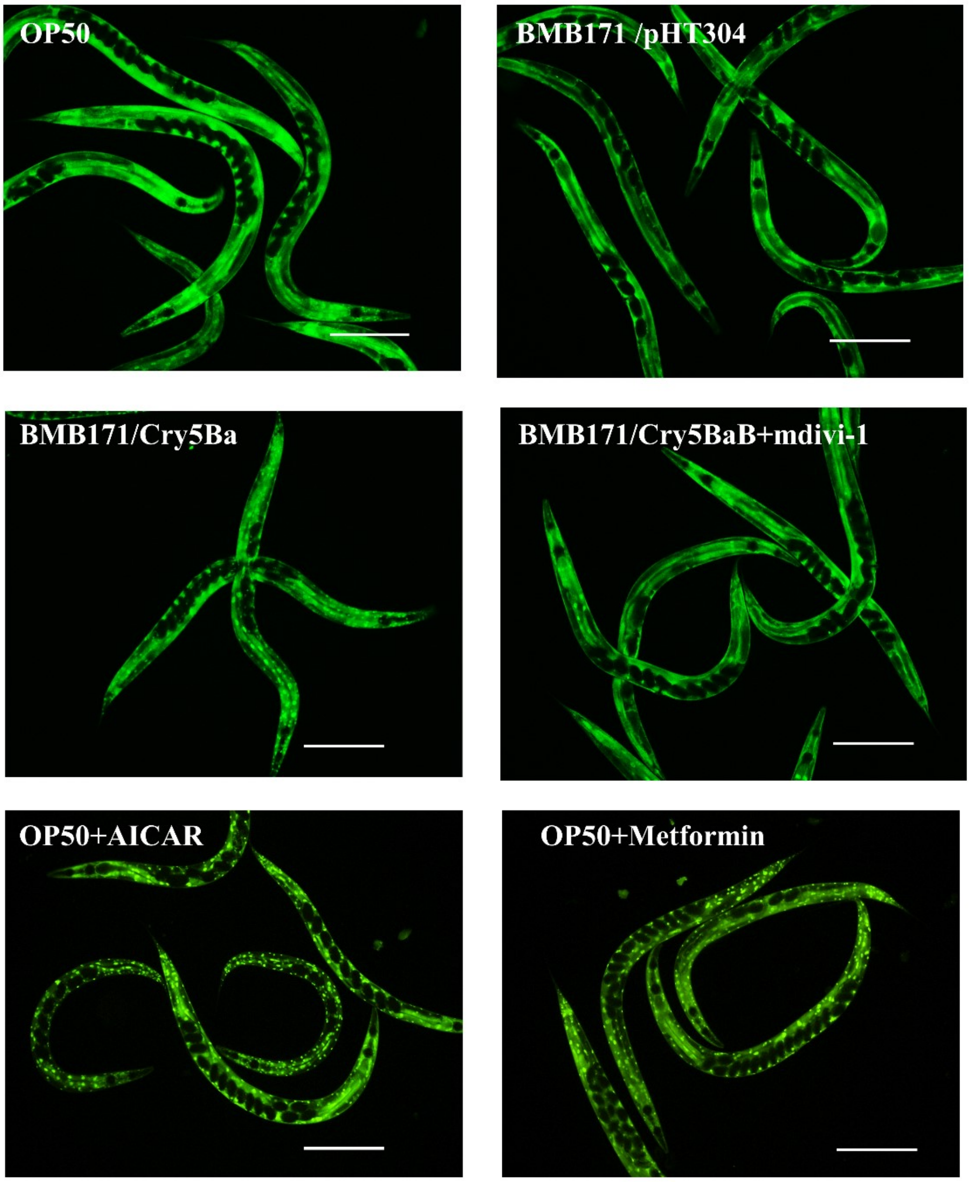

Supplementary Figure 15. DAF-16 nuclear translocation can be significantly influenced by mdivi-1, AICAR and metformin. DAF-16 translocation was observed using the transgenic worms TJ356*(Isdaf-16*::*gfp*) as a reporter. The transgenic worms were fed with OP50, BMB171/Cry5Ba, BMB171/pHT304 or OP50 mixed AICAR, OP50 mixed metformin and BMB171/Cry5Ba mixed with mdivi-1 for 2 h, respectively. The DAF-16::GFP expression pattern was observed using fluorescent microscope (Olympus BX31, Japan) at 40×magnification. Each experiment repeated at least three times and the representative images for each treatment are shown. Scale bar, 20 μm.

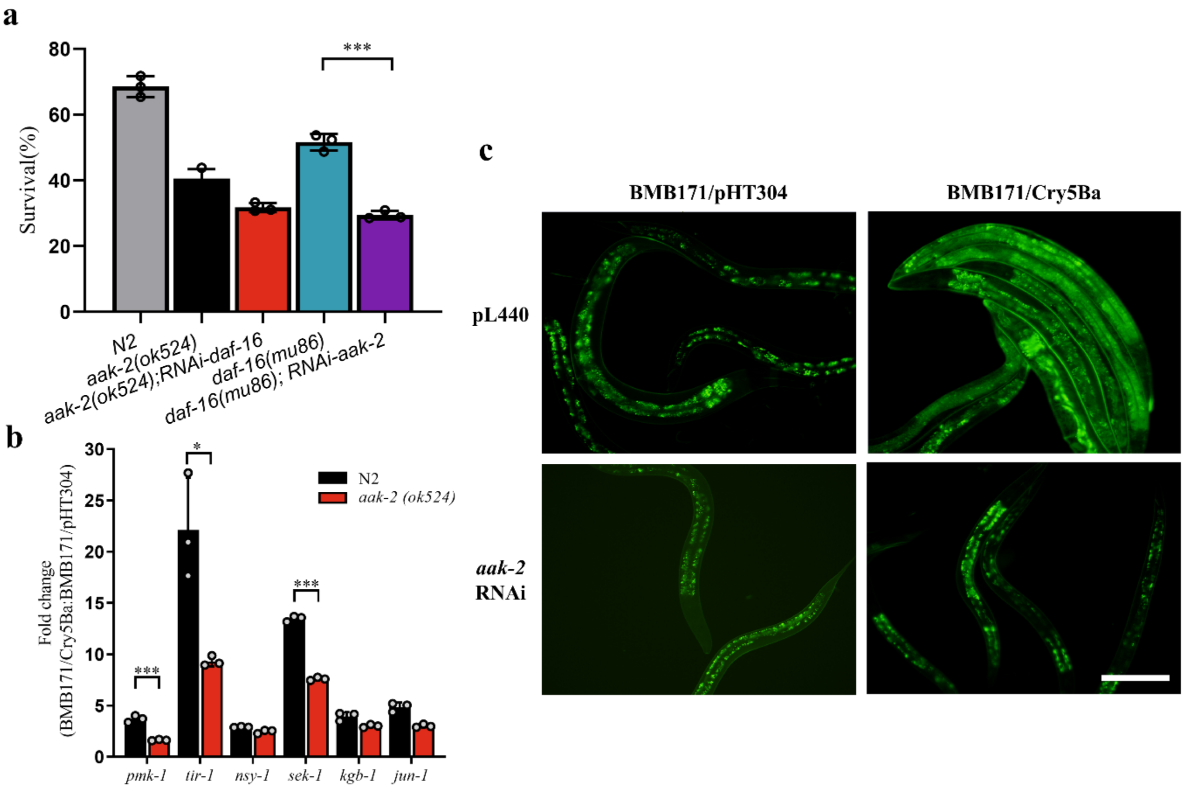

Supplementary Figure 16. The innate immunity p38 MAPK pathway was triggered via AMPK when *C. elegans* fed with BMB171/Cry5Ba. a The alive rates of the wild worm N2, null allele *aak-2* mutant *aak-2 (ok524), aak-2(ok524)/RNAi-daf-16* (reduced the transcription levels of *daf-16* gene in *aak-2* mutant *aak-2 (ok524)),* null allele *daf-16* mutant *daf-16(mu86)* and *daf-16(mu86)/RNAi-aak-2* (reduced the transcription levels of *aak-2* gene in *daf-16* mutant *daf-16(mu86)*) when fed with BMB171/Cry5Ba (20μg/ml toxin Cry5Ba for 60 hours). **b** The transcription levels of MAPK genes *pmk-1, tir-1, nsy-1, sek-1, kgb-1* and *jun-1* of wild type N2 worms and null allele *aak-2* mutant *aak-2 (ok524)* when fed with BMB171/Cry5Ba and BMB171/pHT304. Data represent three biological replicates of representative results from three independent experiments with the same trend. **c** The PMK-1 expression of transgene worms PRJ112 [*pmk1::gfp*] fed with BMB171/Cry5Ba and BMB171/pHT304 were observed using fluorescent microscope; when gene *aak-2* was silenced by RNA interference (RNAi)

technique in transgene worms PRJ112 [*pmk1::gfp*], the PMK-1 expression of the PRJ112 [*pmk-1::gfp*] worms fed with BMB171/Cry5Ba and BMB171/pHT304. N =3 independent experiments. Data points represent the mean values of three independent replicates, error bars denote the SD. The scale bar is 0.02 mm. The p value was determined by a Student’s t-test (Welch's correction for unequal variances). ***: $p < 0.001$, **: $p < 0.01$, *: $p < 0.05$.

Supplementary Tables:

Supplementary Data 1. Source data for Figures. This table shows all of the raw data for the figures.. This table was submitted directly as an Excel file (designated Supplementary Data 1) because it contains a large amount of data that cannot be easily shown in this file.

Supplementary Data 2. Transcriptome analysis data of *C. elegans* after infection of Bt strains. This table was submitted directly as an Excel file (designated Supplementary Data 2) because it contains a large amount of data that cannot be easily shown in this file.

Supplementary Data 3. Enrichment pathway analyses data of *C. elegans* after infection by Bt strains. This table was submitted directly as an Excel file (designated Supplementary Data 3) because it contains a large amount of data that cannot be easily shown in this file.

Supplementary Data 4. Transcriptome analysis data of the genes responded to nematocidal Bt infection compared to AMPK. This table was submitted directly as an Excel file (designated Supplementary Data 4) because it contains a large amount of data that cannot be easily shown in this file.

Supplementary Data 5. Transcriptome analysis data of the genes responded to nematocidal Bt infection compared to DAF-16. This table was submitted directly as an Excel file (designated Supplementary Data 5) because it contains a large amount of data that cannot be easily shown in this file.

Supplementary Data 6. Statistical analysis data of each graph. This table shows the process and results of all statistical analysis in the paper. This table was submitted directly as an Excel file (designated Supplementary Data 6) because it contains a large amount of data that cannot be easily shown in this file.

Supplementary Table 1. Strains and plasmids used in this study.

Strains or plasmids	Characteristics	Source or reference
Bacillus thuringiensis		
BMB171	No plasmid mutant, acrystalliferous Bt strain	[1]
BMB171/pHT304	Derivative of BMB171 containing empty plasmid pHT304 as a control.	Our laboratory
BMB171/Cry5Ba	Derivative of BMB171 containing cry5Ba gene for producing Cry5Ba.	[2]
BMB171/Cry5Ca	Derivative strain of BMB171 containing cry5Ca gene expressing Cry5Ca protein.	[3]
BMB171/Cry6Aa	Derivative strain of BMB171 containing cry6Aa gene expressing Cry6Aa protein.	[2]
BMB171/Cry21Aa	Derivative strain of BMB171 containing cry21A gene expressing Cry21A protein.	[4]
BMB171/Cry1Ac	Derivative of BMB171 containing cry1Ac gene for producing Cry1Ac.	[5]
Escherichia coli		
OP50	An uracil auxotroph strain whose growth is limited on Nematode Growth Medium (NGM) plates	*Caenorhabditis* Genetics Center
HT115	An RNAi feeding strain with tetracycline resistance used for nematode interference experiments.	Gift from Professor Wu Zhengxin
HT115/pL440	*E. coli* HT115 containing empty plasmid pL440.	Gift from Professor Wu Zhengxin
HT115/bre-5	*E. coli* HT115 cloned target gene fragment of *bre-5* as a RNAi strain.	Gift from Professor Wu Zhengxin
HT115/aak-2	*E. coli* HT115 cloned target gene fragment of *aak-2* as a RNAi strain.	Gift from Professor Wu Zhengxin
HT115/daf-16	*E. coli* HT115 cloned target gene fragment of *daf-16* as a RNAi strain.	Gift from Professor Wu Zhengxin
HT115/lys-7	*E. coli* HT115 cloned target gene fragment of *lys-7* as a RNAi strain.	This study

HT115/F32A5.4	*E. coli* HT115 cloned target gene fragment of *F32A5.4* as a RNAi strain.	This study
HT115/tre-4	*E. coli* HT115 cloned target gene fragment of *tre-4* as a RNAi strain.	This study
HT115/sod-3	*E. coli* HT115 cloned target gene fragment of *sod-3* as a RNAi strain.	This study
HT115/clec-166	*E. coli* HT115 cloned target gene fragment of *clec-166* as a RNAi strain.	This study
HT115/thn-2	*E. coli* HT115 cloned target gene fragment of *thn-2* as a RNAi strain.	This study
HT115/ttr-44	*E. coli* HT115 cloned target gene fragment of *ttr-44* as a RNAi strain.	This study
***Caenorhabditis elegans* strains**		
N2	wild type strains	*Caenorhabditis* Genetics Center
HY498	*bre-5*(*ye17*), which was deleted the membrane receptor of Cry5Ba, showing resistance to Cry5Ba.	*Caenorhabditis* Genetics Center
SJ4143	*zcIs17*(P_{ges-1}::GFP^{mt}), which stably expressed GFP in mitochondria matrix of intestinal cells.	*Caenorhabditis* Genetics Center
tm1944	*aak-1*(*tm1944*), which was the null alleles mutant of AMPK subunit α1.	*Caenorhabditis* Genetics Center
RB754	aak-2(ok524), which was the null alleles mutant of AMPK subunit α2.	*Caenorhabditis* Genetics Center
tm2658	aakb-1(tm2658), which was the null alleles mutant of AMPK subunit β1.	*Caenorhabditis* Genetics Center
tm5269	aakg-4(tm5269), which was the null alleles mutant of AMPK subunit γ1.	*Caenorhabditis* Genetics Center
TG38	aak-2(gt33), which was the null allele mutant of *aak-2*.	*Caenorhabditis* Genetics Center
CF1038	*daf-16*(*mu86*), which was the null allele mutant of *daf-16*.	*Caenorhabditis* Genetics Center
PRJ112	*mutEx70*(pmk1::gfp), which expressed GFP by fusing GFP on PMK, working as PMK-1 reporter worm.	*Caenorhabditis* Genetics Center

TJ356	*zIs356*(*Isdaf-16:: gfp*), which expressed GFP by fusing GFP on Daf-16, working as Daf-16 reporter worm.	*Caenorhabditis* Genetics Center
CF1553	*muIs84*(sod-3::GFP), which expressed GFP by fusing GFP on Sod-3, working as Sod-3 reporter worm.	*Caenorhabditis* Genetics Center
RT311	*pwIs69*(P_{vha-6}::GFP::RAB-11), which stably expressed GFP in apical recycling endosome.	*Caenorhabditis* Genetics Center
-	*aak-2*(*ok524*) (P_{vha-6}::*aak-2*), which was a rescued worm that stably expressed *aak-2* in the intestine.	This study
-	*aak-2(ok524)* (P_{myo-3}::*aak-2*), which was a rescued worm that stably expressed *aak-2* in the muscle.	This study
-	*aak-2(ok524)* (P_{rab-3}::*aak*-2), which was a rescued worm that stably expressed *aak-2* in the neuron.	This study
	aak-2(ok524) (P_{aak-2}::*aak*-2), which was the native rescued worm that stably expressed *aak-2*.	This study
**Plasmid**		
pHT304	*E. coil* to *B. thuringiensis* shuttle vector with 4 copies per cell, Amp^r, Erm^r, 6.6 kb.	[6]
pL4440	Vector used for the expression of dsRNA for RNAi in nematodes.	Andrew Fire (Addgene plasmid # 1654)
pPD49.26	Vector for overexpression of cloned tissue-specific promoter.	Andrew Fire (Addgene plasmid # 1686)

Supplementary Table 2. Primer sequences used in this study.

Primers	Sequence (5'→3')	Functions
mtDNA Forward	GTTTATGCTGCTGTAGCGTG	To quantify the content of mtDNA.
mtDNA Reverse	CTGTTAAAGCAAGTGGACGAG	To quantify the content of mtDNA.
ama-1 Forward	TGGAACTCTGGAGTCACACC	To quantify the content of genomic DNA.
ama-1 Reverse	CATCCTCCTTCATTGAACGG	To quantify the content of genomic DNA.
Pvha-6 Forward	CCCAAGCTTTCGACGGCCCGGGCTGGTAAAG	To amplify the upstream of the *vha-6* promoter.
Pvha-6 Reverse	CGCGGATCCTTTGGCGGAGGGACTCAGATGG	To amplify the downstream of the *vha-6* promoter.
Pmyo-3 Forward	CCCAAGCTTGGGCTGCAGGTCGGCTATAATA	To amplify the upstream of the *myo-3* promoter.
Pmyo-3 Reverse	CGCGGATCCTCTAGATGGATCTAGTGGTCGT	To amplify the downstream of the *myo-3* promoter.
Prab-3 Forward	CCCAAGCTTAGTCTTTTAAACAAAAACCCAAA	To amplify the upstream of the *rab-3* promoter.
Prab-3 Reverse	CGCGGATCCTGTGAAATCCGATAGGCAAG	To amplify the downstream of the *rab-3* promoter.
Paak-2 Forward	CCCAAGCTTTAAGGTTTCCGACACGCTCG	To amplify the upstream of the *aak-2* promoter.
Paak-2 Reverse	CGCGGATCCTGAAACAGTATATGCGTTTTAT	To amplify the downstream of the *aak-2* promoter.
F-aak-2 Forward	GGATCCATGTTTTCTCATCAAGATCG	To amplify the upstream of the full-length of *aak-2*.
F-aak-2 Reverse	TTAACGAGCCAGTGTTCCAATC	To amplify the downstream of the full-length of *aak-2*.
U-aak-2 Forward	AGTCATTTTGTATGCACTTCT	To amplify the upstream including the 3-UTR of *aak-2* by overlap PCR.
U-aak-2 Reverse	GGATCCTGGATCTTTCTGAATTTGTTTT	To amplify the downstream including the 3-UTR of *aak-2* by overlap PCR.
lys-7 RNAi Forward	CCCAAGCTTGGTTCCCCCGATTGTTGACT	To amplify the gene fragment of *lys-7* for RNAi strain.
lys-7 RNAi Reverse	CCCAAGCTTTGGGTTGTATGCACGAACGA	To amplify the gene fragment of *lys-7* for RNAi strain.
thn-2 RNAi Forward	CCCAAGCTTCGGCTGGACAATCTAGAAACATT	To amplify the gene fragment of *thn-2* for RNAi strain.
thn-2 RNAi Reverse	CCCAAGCTTTTGCATTGCTCCGAGTTTCTG	To amplify the gene fragment of *thn-2* for RNAi strain.
clec-166 RNAi Forward	CCCAAGCTTTCACAGACTTTGAAAGCGCC	To amplify the gene fragment of *clec-166* for RNAi strain.
clec-166 RNAi Reverse	CCCAAGCTTCAGTCGTCAGGGTAACGCTT	To amplify the gene fragment of *clec-166* for RNAi strain.
sod-3 RNAi Forward	CCCAAGCTTTCTCCAACCAGCGCTGAAAT	To amplify the gene fragment of *sod-3* for RNAi strain.
sod-3 RNAi Reverse	CCCAAGCTTGAACCGAAGTCGCGCTTAAT	To amplify the gene fragment of *sod-3* for RNAi strain.

F32A5.4 RNAi Forward	CCCAAGCTTTGGACAATACGCTCGTGACC	To amplify the gene fragment of *F32A5.4* for RNAi strain.
F32A5.4 RNAi Reverse	CCCAAGCTTAGAGGCCTTGAACTTGGCTT	To amplify the gene fragment of *F32A5.4* for RNAi strain.
ttr-44 RNAi Forward	CCCAAGCTTTGATAAGTACATTCCCGGCCA	To amplify the gene fragment of *ttr-44* for RNAi strain.
ttr-44 RNAi Reverse	CCCAAGCTTCTGTTTTGGGTGATGACGGC	To amplify the gene fragment of *ttr-44* for RNAi strain.
tre-4 RNAi Forward	CCCAAGCTTCCGTGGCCTTTGAAGAAGGA	To amplify the gene fragment of *tre-4* for RNAi strain.
tre-4 RNAi Reverse	CCCAAGCTTTGCAGCAAGTTTTCTGTGGC	To amplify the gene fragment of *tre-4* for RNAi strain.
tba-1 Forward	TCAACATGCCATCGCCGCC	To analysis the transcription of *tab-1* mRNA in *C. elegans.*
tba-1 Reverse	TCCAAGCGAGCCAGGCTTCAG	To analysis the transcription of *tab-1* mRNA in *C. elegans.*
lys-7 Forward	AGGTTCCCCCGATTGTTGAC	To analysis the transcription of *lys-7* mRNA in *C. elegans.*
lys-7 Reverse	GCTGGGTTGTATGCACGAAC	To analysis the transcription of *lys-7* mRNA in *C. elegans.*
F32A5.4 Forward	CAGAGCGAGTTGGCCACTTA	To analysis the transcription of *F32A5.4* mRNA in *C. elegans.*
F32A5.4 Reverse	CATTTTGCAGCCACCACACA	To analysis the transcription of *F32A5.4* mRNA in *C. elegans.*
tre-4 Forward	GGCCAACACTTTGCATTGGT	To analysis the transcription of *tre-4* mRNA in *C. elegans.*
tre-4 Reverse	GGTCGTCCTCGTCGTTTTCT	To analysis the transcription of *tre-4* mRNA in *C. elegans.*
sod-3 Forward	AAATGTCCGCCCAGACTATG	To analysis the transcription of *sod-3* mRNA in *C. elegans.*
sod-3 Reverse	TGGCAAATCTCTCGCTGA	To analysis the transcription of *sod-3* mRNA in *C. elegans.*
clec-166 Forward	GCGCTAATCCAGAGGTCTCC	To analysis the transcription of *clec-166* mRNA in *C. elegans*
clec-166 Reverse	CCCGTATTCGCTACTGGACC	To analysis the transcription of *clec-166* mRNA in *C. elegans*
thn-2 Forward	GAGGCTTCCAACTTACGGCT	To analysis the transcription of *thn-2* mRNA in *C. elegans*
thn-2 Reverse	CAAGGGATGCTGGTGGAACT	To analysis the transcription of *thn-2* mRNA in *C. elegans*
D1086.3 Forward	TGCAGGGATGGCAGAAGTTT	To analysis the transcription of *D1086.3* mRNA in *C. elegans*
D1086.3 Reverse	GGAATGGGTACTCAACCGGA	To analysis the transcription of *D1086.3* mRNA in *C. elegans*
ttr-44 Forward	TCCAGAAAACGCCGTCATCA	To analysis the transcription of *ttr-44* mRNA in *C. elegans*
ttr-44 Reverse	TGAGTAAGCAGTGGCAAAAGG	To analysis the transcription of *ttr-44* mRNA in *C. elegans*
pmk-1 Forward	TGCCCGTCGTACATATCGTG	To analysis the transcription of *pmk-1* mRNA in *C. elegans*
pmk-1 Reverse	GGTCATCGTTGAGTCGCTGA	To analysis the transcription of *pmk-1* mRNA in *C. elegans*
tir-1 Forward	TCCACTTGGACGAACTGACG	To analysis the transcription of *tir-1* mRNA in *C. elegans*

tir-1 Reverse	GCTGGCTGAGTAGGACAAGG	To analysis the transcription of *tir-1* mRNA in *C. elegans*
nsy-1 Forward	TGCTTGCGTGTAGAACTTTTCA	To analysis the transcription of *nsy-1* mRNA in *C. elegans*
nsy-1 Reverse	TGATGATTCGGATGTGTGTGAGT	To analysis the transcription of *nsy-1* mRNA in *C. elegans*
sek-1 Forward	GACACTGTTTGGCGACGATG	To analysis the transcription of *sek-1* mRNA in *C. elegans*
sek-1 Reverse	ATTCCGTCCACGTTGCTGAT	To analysis the transcription of *sek-1* mRNA in *C. elegans*
kgb-1 Forward	ATGGAAGTGGATCTGCCGGT	To analysis the transcription of *kgb-1* mRNA in *C. elegans*
kgb-1 Reverse	AGGTCATCGGCCATTACGAC	To analysis the transcription of *kgb-1* mRNA in *C. elegans*
jun-1 Forward	CAAAAAGGATCGGCGACAGG	To analysis the transcription of *jun-1* mRNA in *C. elegans*
jun-1 Reverse	AGATGCCGGTGTGATTGGAC	To analysis the transcription of *jun-1* mRNA in *C. elegans*

Supporting References:

1 He, J. *et al.* Complete genome sequence of *Bacillus thuringiensis* mutant strain BMB171. *J Bacteriol* **192**, 4074-4075, (2010).

2 Guo, S. *et al.* New strategy for isolating novel nematicidal crystal protein genes from *Bacillus thuringiensis* strain YBT-1518. *Appl Environ Microbiol* **74**, 6997-7001 (2008).

3 Geng, C. *et al.* Dissimilar Crystal Proteins Cry5Ca1 and Cry5Da1 Synergistically Act against Meloidogyne incognita and Delay Cry5Ba-Based Nematode Resistance. *Appl. Environ. Microbiol.* **83**, e03505 (2017)

4 Wan, L. *et al. Bacillus thuringiensis* targets the host intestinal epithelial junctions for successful infection of *Caenorhabditis elegans*. *Environ Microbiol* **21**, 1086-1098 (2019).

5 Fang, S. *et al. Bacillus thuringiensis* bel protein enhances the toxicity of Cry1Ac protein to Helicoverpa armigera larvae by degrading insect intestinal mucin. *Appl Environ Microbiol* **75**, 5237-5243 (2009).

6 Arantes, O. & Lereclus, D. Construction of cloning vectors for *Bacillus thuringiensis*. *Gene* **108**, 115-119 (1991).
