# Supplementary material for: C. elegans monitor energy status via the AMPK pathway to trigger innate immune responses against bacterial pathogens
Source: Commun Biol. 2022 Jun 30;5:643. doi: 10.1038/s42003-022-03589-1 (PMC9246835; doi:10.1038/s42003-022-03589-1)
Supplement: Supplementary file 3 — Description of Additional Supplementary Files [file 42003_2022_3589_MOESM3_ESM.pdf]

Description of Additional Supplementary Files

Supplementary Data 1. Source data for Figure 1-7. This table shows all of the raw data for the figures.. This table was submitted directly as an Excel file (designated **Supplementary Data 1**) because it contains a large amount of data that cannot be easily shown in this file.

Supplementary Data 2. Transcriptome analysis data of *C. elegans* after infection of Bt strains. This table was submitted directly as an Excel file (designated **Supplementary Data 2**) because it contains a large amount of data that cannot be easily shown in this file .

Supplementary Data 3. Enrichment pathway analyses data of *C. elegans* after infection by Bt strains. This table was submitted directly as an Excel file (designated **Supplementary Data 3**) because it contains a large amount of data that cannot be easily shown in this file.

Supplementary Data 4. Transcriptome analysis data of the genes responded to nematocidal Bt infection compared to AMPK. This table was submitted directly as an Excel file (designated **Supplementary Data 4**) because it contains a large amount of data that cannot be easily shown in this file.

Supplementary Data 5. Transcriptome analysis data of the genes responded to nematocidal Bt infection compared to DAF-16. This table was submitted directly as an Excel file (designated **Supplementary Data 5**) because it contains a large amount of data that cannot be easily shown in this file.

Supplementary Data 6. Statistical analysis data of each graph. This table shows the process and results of all statistical analysis in the paper. This table was submitted directly as an Excel file (designated **Supplementary Data 6**) because it contains a large amount of data that cannot be easily shown in this file.
