# Supplementary material for: C. elegans monitor energy status via the AMPK pathway to trigger innate immune responses against bacterial pathogens
Source: Commun Biol. 2022 Jun 30;5:643. doi: 10.1038/s42003-022-03589-1 (PMC9246835; doi:10.1038/s42003-022-03589-1)
Supplement: Supplementary file 10 — Reporting Summary [file 42003_2022_3589_MOESM10_ESM.pdf]

nature portfolio

Corresponding author(s): Donghai Peng, Ming Sun

Last updated by author(s): Apr 29, 2022

Reporting Summary

Nature Portfolio wishes to improve the reproducibility of the work that we publish. This form provides structure for consistency and transparency in reporting. For further information on Nature Portfolio policies, see our Editorial Policies and the Editorial Policy Checklist.

Statistics

For all statistical analyses, confirm that the following items are present in the figure legend, table legend, main text, or Methods section.

n/a	Confirmed	
☐	☒	The exact sample size (*n*) for each experimental group/condition, given as a discrete number and unit of measurement
☐	☒	A statement on whether measurements were taken from distinct samples or whether the same sample was measured repeatedly
☐	☒	The statistical test(s) used AND whether they are one- or two-sided<br>*Only common tests should be described solely by name; describe more complex techniques in the Methods section.*
☒	☐	A description of all covariates tested
☐	☒	A description of any assumptions or corrections, such as tests of normality and adjustment for multiple comparisons
☐	☒	A full description of the statistical parameters including central tendency (e.g. means) or other basic estimates (e.g. regression coefficient) AND variation (e.g. standard deviation) or associated estimates of uncertainty (e.g. confidence intervals)
☐	☒	For null hypothesis testing, the test statistic (e.g. *F*, *t*, *r*) with confidence intervals, effect sizes, degrees of freedom and *P* value noted<br>*Give P values as exact values whenever suitable.*
☒	☐	For Bayesian analysis, information on the choice of priors and Markov chain Monte Carlo settings
☒	☐	For hierarchical and complex designs, identification of the appropriate level for tests and full reporting of outcomes
☒	☐	Estimates of effect sizes (e.g. Cohen's *d*, Pearson's *r*), indicating how they were calculated

Our web collection on statistics for biologists contains articles on many of the points above.

Software and code

Policy information about availability of computer code

Data collection	Densitometric analysis of fluorescence pictures and Western blot were performed using Image Pro Plus ver 6.0.<br>Analysis of RNA-sequencing and transcriptome date were performed by public methods. De-novo assembly with Trinity (https://github.com/trinityrnaseq/trinityrnaseq/wiki), a TGICL (http://sourceforge.net/projects/tgicl/files/tgicl%20v2.1/) was used to further assemble all the unigenes . Sequencing reads were annotated and aligned using Tophat2.<br>Experimental datas were performed using GraphPad Prism (version 9.1; GraphPad Software, La Jolla, California) or Origin (2019b, OriginLab)as curve-fitting program.
Data analysis	All data analysis was performed using SPSS, ver20.0 (SPSS, Chicago, IL, USA) and GraphPad Prism (version 9.1; GraphPad Software, La Jolla, California)

For manuscripts utilizing custom algorithms or software that are central to the research but not yet described in published literature, software must be made available to editors and reviewers. We strongly encourage code deposition in a community repository (e.g. GitHub). See the Nature Portfolio guidelines for submitting code & software for further information.

Data

Policy information about availability of data

All manuscripts must include a data availability statement. This statement should provide the following information, where applicable:

- Accession codes, unique identifiers, or web links for publicly available datasets
- A description of any restrictions on data availability
- For clinical datasets or third party data, please ensure that the statement adheres to our policy

mRNA-sequencing data are available on the NCBI Sequence Read Archive (SRA) (https://www.ncbi.nlm.nih.gov/sra), under the bioproject PRJNA662857 (https://dataview.ncbi.nlm.nih.gov/object/PRJNA662857?reviewer=dnhoor4a4e7johdlqepcm0d53h).

Field-specific reporting

Please select the one below that is the best fit for your research. If you are not sure, read the appropriate sections before making your selection.

☒ Life sciences ☐ Behavioural & social sciences ☐ Ecological, evolutionary & environmental sciences

For a reference copy of the document with all sections, see nature.com/documents/nr-reporting-summary-flat.pdf

Life sciences study design

All studies must disclose on these points even when the disclosure is negative.

Sample size	No sample size has been predetermined in this study.
Data exclusions	No date has been excluded in any data analysis.
Replication	At least three biological replicates are performed for each biological experiment. The semi-quantitative detection by LC/MS is performed on at least three samples for three repetitions.
Randomization	Fluorescence photographed nematodes are randomly photographed for randomized measurement and statistics.
Blinding	Not relevant to the study.

Reporting for specific materials, systems and methods

We require information from authors about some types of materials, experimental systems and methods used in many studies. Here, indicate whether each material, system or method listed is relevant to your study. If you are not sure if a list item applies to your research, read the appropriate section before selecting a response.

Materials & experimental systems

n/a	Involved in the study	
☐	☒	Antibodies
☒	☐	Eukaryotic cell lines
☒	☐	Palaeontology and archaeology
☐	☒	Animals and other organisms
☒	☐	Human research participants
☒	☐	Clinical data
☒	☐	Dual use research of concern

Methods

n/a	Involved in the study	
☒	☐	ChIP-seq
☒	☐	Flow cytometry
☒	☐	MRI-based neuroimaging

Antibodies

Antibodies used	AMPKα2 Rabbit, Cell Signaling, #2757<br>Phospho-AMPKα(Thr172) Rabbit mAb, Cell Signaling, # 2535<br>β-actin Antibody, Proteintech, # 66009
Validation	AMPKα2 Rabbit, Cell Signaling, #2757<br>Validated through western blot analysis of extracts from HEK293 and COS cells using AMPKα2 Antibody. Relevant citations:Hardie, D.G. (2004) J Cell Sci 117, 5479-87; Carling, D. (2004) Trends Biochem Sci 29, 18-24; Lizcano, J.M. et al. (2004) EMBO J 23, 833-43; Shaw, R.J. et al. (2004) Proc Natl Acad Sci USA 101, 3329-35; Woods, A. et al. (2003) J Biol Chem 278, 28434-42.<br><br>Phospho-AMPKα(Thr172) Rabbit mAb, Cell Signaling, # 2535<br>Validated through western blot analysis of extracts from untreated or oligomycin-treated (0.5 μM) C2C12 cells using Phospho-AMPKα

(Thr172) (40H9) Rabbit mAb.Relevant citations:Hardie, D.G. (2004) J Cell Sci 117, 5479-87; Carling, D. (2004) Trends Biochem Sci 29, 18-24; Lizcano, J.M. et al. (2004) EMBO J 23, 833-43;Shaw, R.J. et al. (2004) Proc Natl Acad Sci USA 101, 3329-35; Woods, A. et al. (2003) J Biol Chem 278, 28434-42; Kim, E.K. et al. (2004) J Biol Chem 279, 19970-6; Hadad, S.M. et al. (2009) BMC Cancer 9, 307; β-actin Antibody, Proteintech, # 66009.

β-actin Antibody, Proteintech, # 66009
Validated through A549 cells (shcontrol and shRNA of Beta Actin) subjected to SDS PAGE followed by western blot with 66009-1-Ig.Relevant citations:Damaris N Lorenzo et al. (2017) Proc Natl Acad Sci U S A. 28;114(48); Yongxuan Yao et al. (2019) J Virol. 5;93(6):e02161-18; Zeyang Ji et al. (2019) Cell Rep. 19;26(8):2064-2077.

Animals and other organisms

Policy information about studies involving animals; ARRIVE guidelines recommended for reporting animal research

Laboratory animals	Caenorhabditis elegans from Caenorhabditis Genetics Center (CGC) or the National Bioresource Project (NBRP)
Wild animals	No wild animals were involved in this study.
Field-collected samples	Not relevant to the study.
Ethics oversight	Not relevant to the study.

Note that full information on the approval of the study protocol must also be provided in the manuscript.
